# Detection and perception of generic host volatiles by mosquitoes: responses to CO_2_ constrains host-seeking behaviour

**DOI:** 10.1098/rsos.170189

**Published:** 2017-05-10

**Authors:** Shahid Majeed, Sharon Rose Hill, Teun Dekker, Rickard Ignell

**Affiliations:** Unit of Chemical Ecology, Department of Plant Protection Biology, Swedish University of Agricultural Sciences, PO Box 102, 230 53 Alnarp, Sweden

**Keywords:** carbon dioxide, behaviour, electrophysiology, host recognition

## Abstract

Natural selection has favoured specialization in anthropophilic mosquito host choice, yet in the absence of human hosts, females feed on a selected range of vertebrates. For host recognition, we hypothesize that mosquitoes primarily rely on generic host volatiles. Detection and perception of such compounds would provide the mosquito with a flexible, yet constrained, odour coding system that could delineate host preference. In this study, we show that the quintessential generic volatile for host-seeking, carbon dioxide, activates and attracts the malaria mosquito, *Anopheles coluzzii*, and the arbovirus vectors, *Aedes aegypti* and *Culex quinquefasciatus*, within boundaries set by the dynamic range and coding capacity of the CO_2_-sensitive olfactory receptor neurons. These boundaries are sufficiently broad to elicit behavioural responses to various hosts within their preferred host range. This study highlights the significance of the sensitivity of the carbon dioxide detection system and its regulation of host seeking and recognition.

## 1. Background

Mosquitoes that transmit infectious diseases often express a marked, inherent host preference [1–6]. Host preference studies of the African malaria vector, *Anopheles gambiae sensu lato*, and the arbovirus vectors, *Aedes aegypti* and *Culex quinquefasciatus*, show that natural selection favours a restricted host breadth [3]. Despite this, there remains sufficient plasticity in host preference to provide a mechanism by which mosquitoes can adapt to different environmental conditions [1,3,5], which is an important variable regulating disease transmission by predominantly anthropophilic mosquitoes [6]. This indicates that there is both a cost and benefit to maintaining plasticity [1] and/or that these species are physiologically limited in the capacity to be plastic.

Olfaction is the principal sense by which mosquitoes locate their hosts [3,7]. Host discrimination and selection is a sequence of behaviours that includes activation, long- and short-range attraction, and landing on the host [8]. Initial recognition of an upwind host relies on the detection of minute fluctuations in carbon dioxide (CO_2_) concentration, which elicits activation and subsequent attraction in host-seeking mosquitoes [8–10], even in the absence of other host odours [8]. Emitted by all vertebrates, CO_2_ also gates the attraction to host odours over a range of distances in host-seeking mosquitoes [11,12]. Observed interspecific variation in the behavioural response to CO_2_ may be attributed to differences in the dynamic range of the CO_2_-chemosensory system for each mosquito species [9,13,14], and the underlying mechanism regulating the CO_2_ dynamic range is one of sensory constraint [15,16].

We hypothesize that such limitations could be generated by a series of constraints on the sensory system used to detect and discriminate between potential host species at different distances, from activation and attraction (this study) to short-range acceptance (see companion paper: [17]). Here, we test the hypothesis that host preference correlates with the receptive range of the CO_2_-chemosensory system, and suggest that the behavioural response of anthropophilic mosquitoes to CO_2_ is constrained by limits in sensory acuity.

## 2. Methods

### 2.1. Insects

*Aedes aegypti* (Rockefeller strain), *Anopheles coluzzii* (Suakoko strain; previously *Anopheles gambiae* M molecular form) and *Culex quinquefasciatus* (Thai strain) were reared at 27 ± 2°C, 70 ± 2% relative humidity (RH) under a 12 h : 12 h light : dark period, as previously described [17,18]. For all experiments, 4- to 10-day post-emergence sugar-fed adult female mosquitoes were used.

### 2.2. Single sensillum recordings

The maxillary palps of *Ae. aegypti*, *An. coluzzii* and *Cx. quinquefasciatus* are covered with capitate peg sensilla, variously described as peg sensilla or basiconic sensilla, each housing three olfactory receptor neurons (ORNs) [16,18–20]. In all species, the ORN with the largest amplitude is, by convention, referred to as the A cell, and has previously been shown to be an absolute detector of CO_2_ below 1200 ppm [15,16,19,20]. Electrophysiological recordings from this neuron were made and analysed as previously described [16].

A continuous humidified stream of synthetic air (Strandmöllen AB, Ljungby, Sweden), lacking CO_2_, was passed over the maxillary palp (2 l min^−1^) via a glass tube (7 mm i.d.). Carbon dioxide was introduced into the air stream through a hole (2 mm i.d.) in the glass tube, 11 cm upstream of the maxillary palps. Delivery of CO_2_ was regulated by two-way Teflon solenoid valves (Teddington, Skogås, Sweden), controlled via the digital output of an IDAC-4 (Syntech, Germany). Each valve was connected to separate gas cylinders containing metered amounts of CO_2_ (150, 300, 600, 1200, 2400, 4800 ppm) and oxygen (20%), balanced by nitrogen (Strandmöllen AB). A pulsed stimulus train of CO_2_ was used, with stimulation for 1 s and an interstimulus interval of 1 s.

### 2.3. Flight tube bioassay

Behavioural responses to pulsed CO_2_ stimuli were assessed in a glass flight tube bioassay (80 × 9.5 cm i.d.), as previously described [16], with minor modifications (figure 3*b*). Briefly, the tube assay was illuminated from above with white light at 280 lux for the diurnal *Ae. aegypti*, while red light (40 lux) was used for the nocturnal *An. coluzzii* and *Cx. quinquefasciatus*. Experiments for each species were conducted during their period of peak host-seeking activity [17]. Charcoal filtered humidified air (25 ± 2°C, RH 65 ± 2%) flowed through the flight tube at 30 cm s^−1^. To ensure a laminar flow and a homogeneous plume structure, the air passed through a series of stainless steel mesh screens prior to entering the flight tube (figure 3*b*). A pulsed flow of pure CO_2_ (Strandmöllen AB), regulated by a stimulus controller (SEC-2/b, Syntech, Germany), was introduced into a pulse generator. Homogenized CO_2_ pulses were delivered, using the same pulsation protocol as for the physiological experiments above, at the desired concentration (600, 1200, 2400 or 4800 ppm) through mixing pure CO_2_ with pressurized air at 4.5 l min^−1^ in the pulse generator, as previously described [16]. The concentration of CO_2_ in the flight tube was measured using a CO_2_ analyser (LI-820, LICOR Biosciences, Lincoln, NE, USA). Controls consisting of exposing individual mosquitoes in the flight tube to non-pulsed ambient CO_2_ levels (385.4 ± 6.28 ppm) that varied minimally over the duration of the experiment (1.3 ± 0.60 ppm) were run daily.

Individual mosquitoes were kept in glass release chambers (7 × 2.6 cm i.d.), covered with stainless steel mesh on one side and a cotton plug on the other, in the bioassay room for 24 h prior to the experiments [16]. The following times were measured: the time after opening the release chamber to take-off (flight activation), the time from take-off to upwind flight directed towards the odour source (halfway, 40 cm), and the time from halfway to source contact. The maximum time recorded was 120 s. Thirty individuals of each species were observed at each concentration of CO_2_. To minimize the effect of daily variation in baseline activity and responses to odours, an equal number of test and control individuals were observed each day.

### 2.4. Statistical analysis

Repeated measures 2-way ANOVA, followed by a Bonferroni post hoc test was performed to compare the physiological activity among the species. The behavioural data were treated in two ways. Two-way ANOVAs, followed by a Tukey post hoc test, were used to compare the time to response among treatments and controls, as well as across species, using GraphPad Prism v. 5.01 for Mac (GraphPad Software, La Jolla California, USA). The number of mosquitoes responding were analysed with nominal logistic regression, comparing treatments and controls for each species and concentration of CO_2_ (JMP^®^, Version 12.0.1, SAS Institute Inc., Cary, NC, 1989--2007).

## 3. Results

### 3.1. Physiological response to carbon dioxide

**Figure 1.**
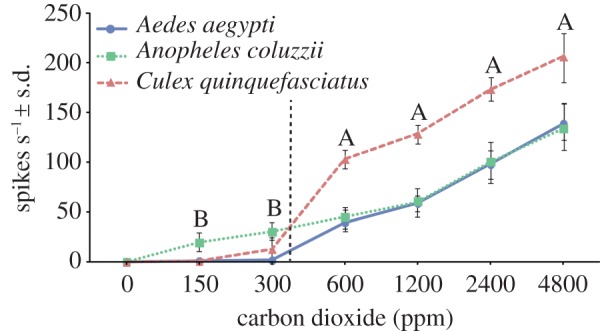
The A cell (*n* = 10) in the maxillary palps of *Aedes aegypti, Anopheles coluzzii* and *Culex quinquefasciatus* is differentially sensitive to carbon dioxide. ‘A’ represents a significant difference in neuronal firing rate in *Cx. quinquefasciatus* compared to *An. coluzzii* and *Ae. aegypti* (*p* < 0.0001); whereas ‘B’ depicts a significant difference in firing in *An. coluzzii* compared to *Ae. aegypti* and *Cx. quinquefasciatus,* respectively (two-way repeated-measures ANOVA, *p* < 0.05). Black dashed line indicates the average ambient CO_2_ concentration.

Stimulation with single pulses of CO_2_ with increasing concentrations elicited a dose-dependent response in the A cell of the capitate peg sensilla of all mosquito species (figure 1). The threshold of neuronal response to CO_2_, in a 0 ppm CO_2_ background, was lowest for *An. coluzzii*. At concentrations of 150 and 300 ppm CO_2_, the ORN activity was significantly higher in *An. coluzzii* than in *Ae. aegypti* and *Cx. quinquefasciatus* (*t* = 3.12, d.f. = 8, *p* < 0.05; *t* = 4.80, d.f. = 8, *p* < 0.001 and *t* = 3.09, d.f. = 8, *p* < 0.05; *t* = 2.92, d.f. = 8, *p* < 0.05, respectively). However, at concentrations exceeding 300 ppm, i.e. above ambient CO_2_ levels (350–400 ppm; indicated in figure 1), the ORN activity in *Cx. quinquefasciatus* was significantly higher than that of the other species (*t* = 10.61, d.f. = 8, *p* < 0.001).

**Figure 2.**
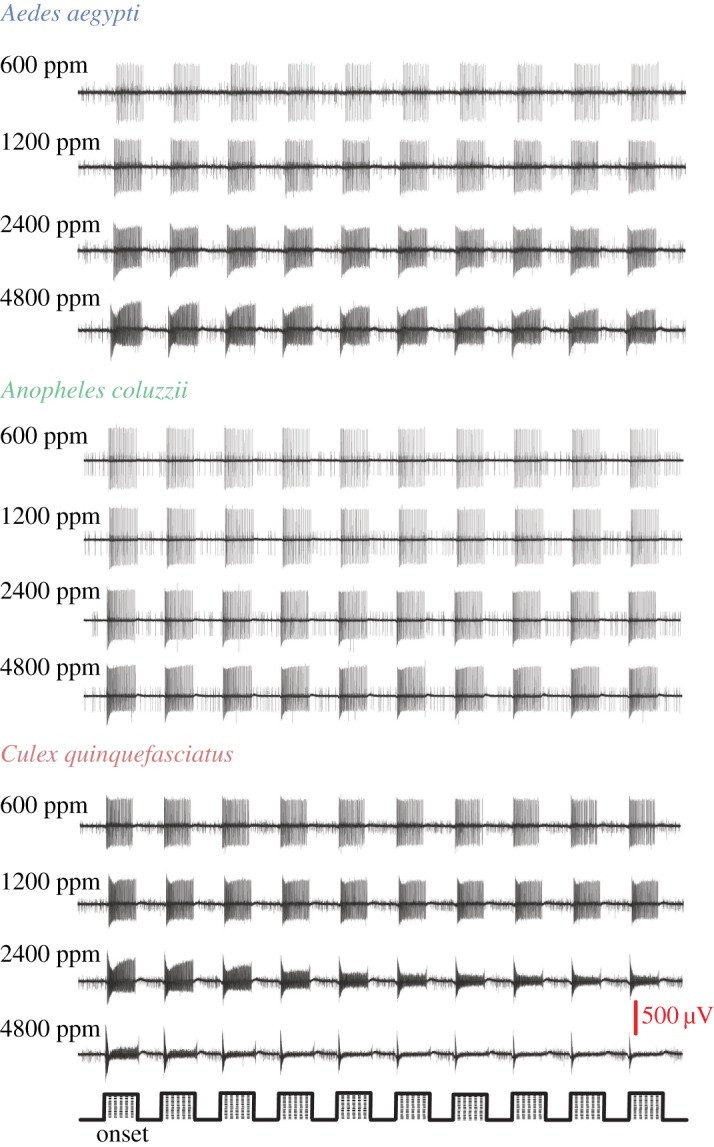
The temporal coding capacity of the CO_2_-sensitive neuron in female *Aedes aegypti, Anopheles coluzzii* and *Culex quinquefasciatus*, over increasing concentrations of CO_2_. The CO_2_ stimuli were delivered in trains of ten pulses, one second on and one second off, as indicated below the response traces (bars). Scale bar indicates spike amplitude (µV).

Pulsed stimuli of CO_2_ induced a phasic-tonic response from the A cell that remained unaltered and dependent on the stimulation. At concentrations of 600 and 1200 ppm CO_2_, the A cell of all species was able to detect and track the pulsed stimuli (figure 2). While, at higher concentrations all species were able to detect the pulses, only *Ae. aegypti* and *An. coluzzii* were able to track the stimuli, i.e. fire in response to CO_2_ throughout each pulse (figure 2). The A cell of *Cx. quinquefasciatus*, while detecting pulse onset rapidly, adapted to each CO_2_ pulse and with subsequent pulses affecting its ability to disadapt, thus limiting its capacity to track the stimuli at the higher concentrations.

### 3.2. Behavioural response to carbon dioxide

**Figure 3.**
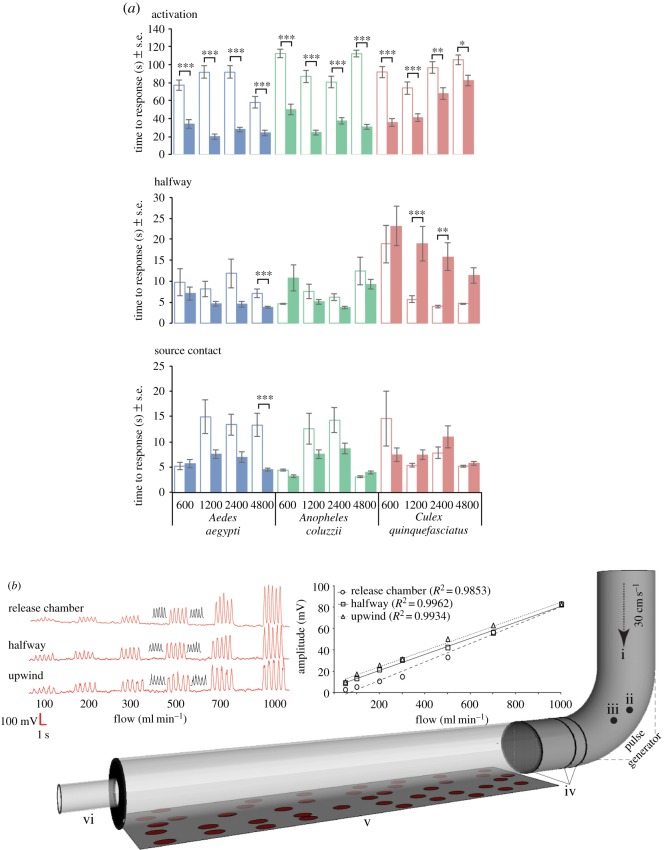
The behavioural response of female *Aedes aegypti, Anopheles coluzzii* and *Culex quinquefasciatus* to pulsed CO_2_ stimuli, over increasing concentrations. (*a*) The time to activation, halfway and source contact of female mosquitoes in the flight tunnel towards constant ambient CO_2_ (control; open bars) and pulsed stimuli of the indicated concentrations of CO_2_ (filled bars; *n* = 30 each species). Asterisks indicate the significant differences among treatments and control (two-way ANOVA; *p < 0.05, **p < 0.01, ***p < 0.001). Vertical bars represent the standard error of means ± SE. (*b*) Behaviour was assessed in a flight tube assay: (i) charcoal-filtered and humidified air, (ii) pressurized air inlet, (iii) stimulus inlet into which CO_2_ was injected, (iv) stainless-steel mesh plume diffusers, (v) glass flight tube, and (vi) release chamber. The upper panels demonstrate that the pulsed stimuli (here shown as five cycles of 1 s on and 1 s off) maintain their amplitude and shape throughout the flight tube and at all tested flow rates. The upper left panel shows the consistent and distinct pulsed stimuli at ascending flow rates of known concentration of acetone in the flight tube. Discrete pulsed stimuli were measured in the centre (in red) and at the lateral sides (in black) of the release chamber, at halfway and at the source. The upper right panel presents a graphical representation of the distinct pulsed stimuli, which shows the average amplitude of each of the five distinct pulses (*N* = 10) at different positions and the regression correlation coefficients (*R*^2^) that demonstrate the consistency of the stimulus amplitude at the different positions within the flight tube with increasing flow rates.

**Figure 4.**
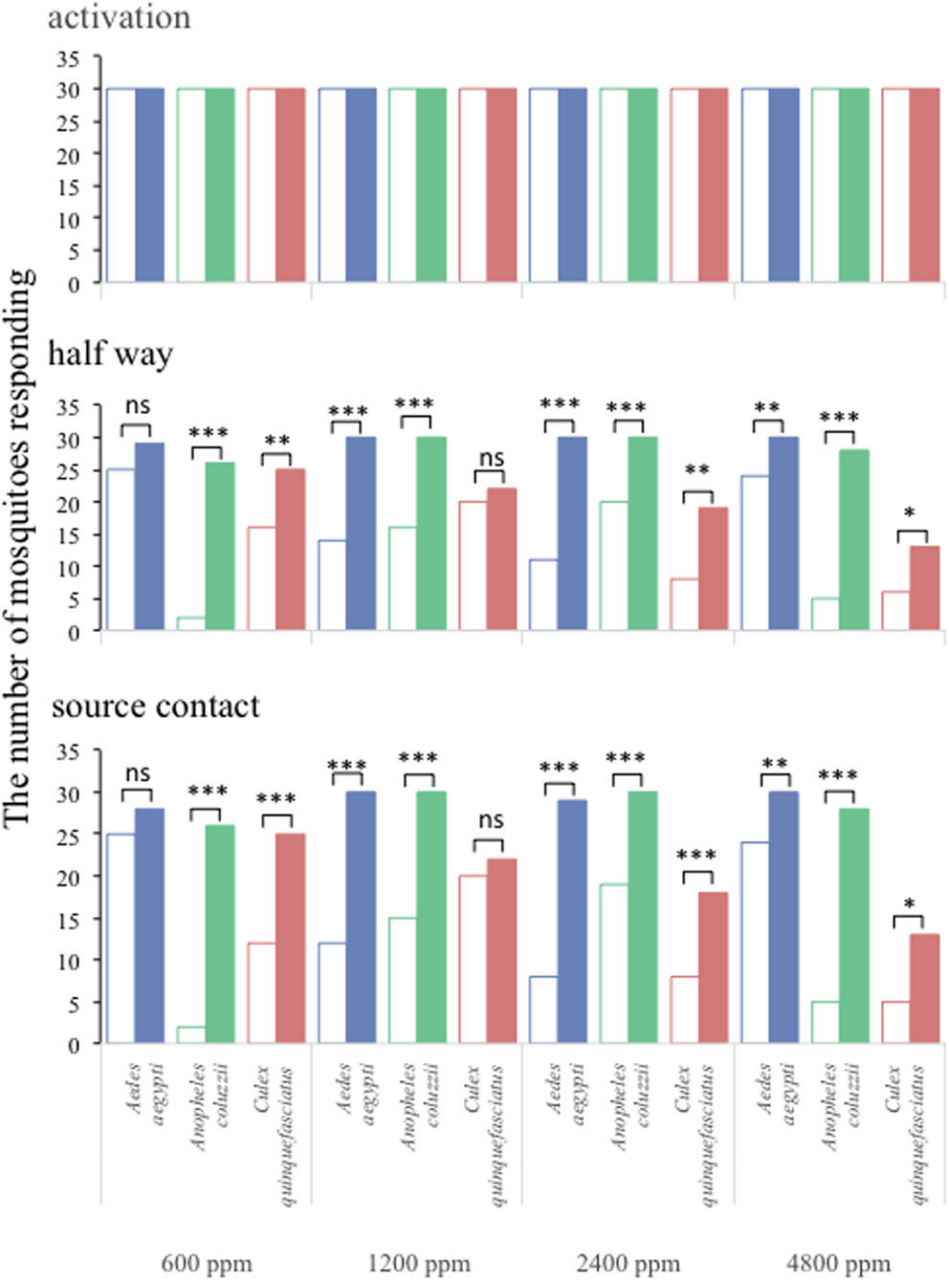
The numbers of female *Aedes aegypti, Anopheles coluzzii* and *Culex quinquefasciatus* responding to constant ambient CO_2_ (control; open bars) and pulsed stimuli of the indicated concentrations of CO_2_ (filled bars). Asterisks indicate the significant differences among treatments and control (nominal logistic regression; *p < 0.05, **p < 0.01, ***p < 0.001).

The behavioural responses to pulsed CO_2_ stimuli differed between species. The time to activation decreased in the presence of pulsed CO_2_ compared with the controls (figure 3*a*; upper panel). Comparing among the species, the time to activation was increased in *Cx. quinquefasciatus* compared to *Ae. aegypti* and *An. coluzzii*, at concentrations above 1200 ppm (2400 ppm: *t*-ratio −4.88, *p* < 0.0001; *t*-ratio −8.48, *p* < 0.0001; 4800 ppm: *t*-ratio −3.72, *p* = 0.0035; *t*-ratio −7.53, *p* < 0.001, respectively; figure 3*a*; upper panel). All mosquitoes of each of the three species were activated in both the presence of pulsed CO_2_ and the constant ambient CO_2_ control experiments (figure 4; upper panel).

In response to pulsed CO_2_, the observed trend was a decrease in time to reach halfway along the flight tube (halfway) compared to the controls, for both *Ae. aegypti* and *An. coluzzii* as the concentration of CO_2_ increased, resulting in a significant difference at 4800 ppm for *Ae. aegypti* (figure 3*a*; middle panel). The opposite trend was observed for *Cx. quinquefasciatus*. Females of *Cx. quinquefasciatus* took significantly longer time to reach halfway at 1200 and 2400 ppm compared to controls (figure 3*a*; middle panel). In addition, to reach halfway took significantly less time for *Ae. aegypti* and *An. coluzzii*, compared to *Cx. quinquefasciatus*, at concentrations exceeding 600 ppm CO_2_ (1200 ppm: *t*-ratio −4.72, *p* < 0.0001; *t*-ratio −4.55, *p* = 0.0002; 2400 ppm: *t*-ratio −4.03, *p* = 0.0014; *t*-ratio −4.32, *p* = 0.0005; 4800 ppm: *t*-ratio −3.47, *p* = 0.0098, respectively; figure 3*a*; middle panel), with one exception. At the highest CO_2_ concentration tested, *An. coluzzii* took as long as *Cx. quinquefasciatus* to reach halfway, which was significantly slower than *Ae. aegypti* (4800 ppm: *t*-ratio −3.16, *p* = 0.0248; figure 3*a*; middle panel). In general, the time to make source contact did not differ between the controls and the pulsed CO_2_ stimuli for all three species (figure 3*a*; lower panel). There was one exception, *Ae. aegypti* reached the source faster in the presence of 4800 ppm CO_2_ than to the control (figure 3*a*; lower panel).

In general, the average number of mosquitoes that reached halfway (figure 4; middle panel) and made source contact (figure 4; lower panel) in the flight tube significantly increased in the presence of pulsed CO_2_ as compared with the control experiments. The proportion of mosquitoes reaching halfway in the flight tube was between 93% to 100% of the tested individuals of *Ae. aegypti* and *An. coluzzii* for all concentrations tested, whereas that of *Cx. quinquefasciatus* declined from 100% to 70% as concentration increased (figure 4; middle panel). Similarly, 87% to 100% of *Ae. aegypti* and *An. coluzzii* made source contact after flying upwind to pulsed CO_2_ (figure 4; lower panel), whilst 83% to 43% of the tested *Cx. quinquefasciatus* made source contact to increased concentrations of pulsed CO_2_ (figure 4; lower panel).

## 4. Discussion

Host choice by mosquitoes is, in part, regulated through senses that have been adapted to preferred hosts, and sensory constraint is a mechanism by which host breadth is regulated [7,21]. Here, we support the previous finding that detection and perception of CO_2_ by the olfactory system play a vital role in the activation of host-seeking behaviour [8–16]. The response characteristics of the CO_2_-detecting ORNs differ, however, among mosquito species, correlating with differential behavioural outputs. The data provided here emphasize that CO_2_ affords host recognition cues to mosquitoes, and that the detection and perception of CO_2_ provide mosquitoes with a dynamic, yet constrained, coding system for host finding.

### 4.1. Constraint in detection limits the responsiveness to CO_2_

The physiological and behavioural responses of the studied mosquito species to CO_2_ differed. In *Cx. quinquefasciatus*, the limited sensory ability to continuously respond throughout the pulses of CO_2_ at concentrations exceeding 1200 ppm, approximating that emitted by a large mammal [13], constrained the behavioural response, particularly activation. The concentration at which the first response was detected when stimulated with single pulses, above ambient CO_2_ levels, and the slope of the dose-response curve of the CO_2_-sensitive ORN in *Cx. quinquefasciatus*, indicate that CO_2_ sensing is more acute at lower ecologically relevant concentrations in this species. The increased sensitivity and reduced dynamic range of the CO_2_-sensitive ORNs in *Cx. quinquefasciatus*, compared with *Ae. aegypti* and *An. coluzzii*, when challenged with multiple pulses of high CO_2_ concentrations, correlates with differences in host preference breadth. Whereas all three species feed on human hosts, they also demonstrate plasticity in feeding behaviour. The hosts of *Ae. aegypti* and *An. coluzzii* include a range of mammals, whereas *Cx. quinquefasciatus* shifts between humans and birds, depending on host availability [3]. It appears that the preference of *Cx. quinquefasciatus* for birds has exerted a selective pressure on the CO_2_-chemosensory system to activate in response to and to follow intermittent contacts with CO_2_ filaments, which, because of the size of the birds, are smaller, of lower average concentration and probably rarer [22]. Having such an acute CO_2_-chemosensory system may have put restrictions on the dynamic range of the CO_2_-sensitive ORNs, as the neurons rapidly adapt, and disadapt more slowly, when stimulated with intermittent pulses of CO_2_ at high (greater than or equal to 2400 ppm) concentrations. It is likely that the CO_2_-sensory machinery has become saturated, reducing the sensitivity to repetitive stimulation. Similar restrictions have been described for pheromone-responsive ORNs in moths, showing that the tracking ability of single ORNs and the behavioural response to repeated stimuli is dependent on adaptation-disadaptation mechanics (for review see [23]). The constrained response of ORNs to high CO_2_ concentrations, when provided in multiple pulses, does not impede the activation and attraction of *Cx. quinquefasciatus* to CO_2_ emitted by humans, as they readily enter houses and tents with a sleeping person, where the measured CO_2_ concentration at the entrance of the tents was at or below 1500 ppm [24], which is within the dynamic range of their CO_2_-chemosensory system (this study). However, the time to activation and halfway, described in this study, increased in the presence of CO_2_ at or above 1200 ppm compared to controls, emphasizing that the CO_2_-chemosensory system is constrained at elevated CO_2_ concentrations over the short range. The CO_2_-sensitive ORNs of *Ae. aegypti* and *An. coluzzii* are less acute at concentrations above ambient CO_2_ levels, which is in agreement with previous studies [15,19], indicating that these highly anthropophilic mosquitoes are less dependent on CO_2_ alone in favour of other host cues as a basis for host selection. The dynamic range of their CO_2_-sensitive ORNs is wide, with a threshold at or below ambient concentration (350–400 ppm) and the neurons do not reach their maximal response at the highest dose tested (4800 ppm). Behavioural analysis also shows that both species are activated and attracted to the full concentration range of CO_2_ tested, which is in line with previous reports [14,25]. Thus, the CO_2_-chemosensory systems of *Ae. aegypti* and *An. coluzzii* are equipped to detect a wide range of CO_2_ emissions, from rare and intermittent CO_2_ signals at a distance of several metres from a potential host [26–28] to amounts equivalent to that emitted by a human or other large mammalian host [27]. In conclusion, host selection by *Cx. quinquefasciatus* is constrained by the dynamic range of their CO_2_-sensitive ORNs dictating the behavioural response, in particular activation, of this species. This may define the breadth of intrinsic preference and allow for behavioural plasticity within these limits.

## 5. Conclusion

While the role of CO_2_ in activating and attracting mosquitoes to potential hosts is well characterized [11,12], this study highlights the importance of CO_2_, within natural release rates, in regulating host seeking and recognition. The cross-species comparison revealed the importance of analysing the response properties and tuning of CO_2_-sensitive ORNs together with how this may affect the behavioural output. From a vector control perspective, this is essential when developing lures for optimal attraction of specific mosquito species in efforts to control and monitor populations.

## Data accessibility

All data that are relevant to the study are reported within the article.
